# The Susceptibility of *Candida albicans* to Gamma-Radiations and Ketoco-nazole Depends on Transitional Filamentation

**DOI:** 10.2174/1874285800802010066

**Published:** 2008-05-23

**Authors:** Simone Cagnacci, Rachele Grasso, Anna Marchese, Renzo Corvò, Eugenio Debbia, Lorenzo Rossi

**Affiliations:** 1 Institute of Microbiology “C.A Romanzi”, Department DISCAT, University of Genoa, Largo Rosanna Benzi, 10, 16132, Genoa, Italy; 2 Department of Radiotherapy, National Cancer Institute of Genoa and University of Genoa, Largo Rosanna Benzi, 10, 16132, Genoa, Italy

## Abstract

The virulence of *C. albicans* is associated with the transitional evolution from yeast to filamentous forms. We were interested in the effects amphotericin B (AMB), ketoconazole (KTC) and γ-radiations might have on these broadly defined phenotypes as determined by the CFU procedure. By using collagen gel as the 3-dimensional support of cell culture, diverse experimental conditions were contemplated in order to modulate the differentiation of Candida during sessile and planktonic growth. These conditions included the co-culture with human epithelial and endothelial cells and treatment with farnesol, tyrosol and conditioned medium from *P. aeruginosa*. The overall results were as follows: 1) The survival of Candida was inhibited by the exposure to γ-radiations, but only after the organism was induced to progress into excess filamentation, while in normal growth conditions it proved to be radioresistant; 2) AMB inhibited the growth of yeast forms, while KTC was specifically toxic to filamentous forms and 3) the combined treatment of filamentous Candida with KTC and γ-radiations resulted in the synergistic inhibition of the organism. These findings indicate that both the radiosensitivity of *C. albicans* and its response to the synergistic effects of γ-radiations and KTC are filamentation-dependent pharmacological processes.

## INTRODUCTION

The ability of *C. albicans* to undergo dramatic changes in cellular morphology under a variety of microenvironmental signals has attracted much attention to the effect that this plasticity may represent a major contribution to the virulence of the organism [1]. As a commensal and a pathogen *C. albicans* can growth attached (sessile) to mucosal structures or floating (planktonic) in the bloodstream and other body fluids. In the sessile condition it can mature into biofilms by undergoing reversible morphological transitions from budding yeasts to filamentous forms, both conditions responding to quorum sensing factors *via *specific replicative and metabolic pathways [2]. Yeast forms are regarded as the core cells on which biofilms develop, whereas filamentation comprises complex morphological transitions, of which at least two, pseudohyphae and hyphae, are commonly observed in biofilms and have received a majority of the research focus [3]. In pseudohyphae, the cells are attached end-to-end and each cell has an elliptical shape with constrictions at the septa. In hyphae, these constrictions are absent and a row of cells show a relatively uniform width. Mixed populations of pseudohyphae and hyphae are a common outcome of filament-inducing conditions and biofilms.

While abundant information is available on the role of antimycotic drugs, notably amphotericin B and ketoconazole [4], in the control of *C. albicans*, to date there are few, conflicting reports concerning the effects of ionizing radiations on this fungus. In general, *in vitro* studies have indicated that radiation therapy can either inhibit Candida proliferation or it can potentiate its virulence, the outcome depending on the source of irradiation, Candida strain and culture conditions [5,6]. In any case, there appear to be no published data on the effects of radiations on Candida grown in collagen gel, a knowledge of considerable importance for mucosal and systemic candidiasis.

The difficulty in dealing with *C. albicans* is manifested by its abilility to colonize and cause infections through the timed expression of virulence factors during biofilm maturation, a powerful obstacle facing antimycotic therapies because of the intrinsic resistance of biofilms to a broad spectrum of antifungals in clinical use [7]. At least two conditions that are encountered *in vivo* are expected to contribute to the complexity of host-microbe interactions. They are: 1) Candida interacts directly or indirectly with host cells, an unavoidable consequence of infective commensalism and surely to complicate the outcome of antifungal therapies and 2) Candida adapts quickly to the diverse microenvironments encountered during the invasive infection of organs and tissues, driven by its phenotypic instability as embodied by yeast-to-hyphal reversible transitions.

Considering that the implementation of these points into existing *in vitro* models could improve ways to translate data from the laboratory to the clinic, in the present work Candida cells were grown in collagen gel, alone or together with human cells to reproduce significant aspects of the infective process. By adopting this model, we asked whether certain Candida morphological transitions are more sensitive than others to ionizing radiations and selected antimycotic drugs, an information prospectively useful to overcome the antibiotic resistance of Candida biofilms and to gain insights into the mechanisms of microbe-host interaction. We provide evidence that during filamentation induced by a variety of agents *C. albicans* is particularly sensitive to growth inhibition by ionizing radiations and ketoconazole, and that synergistic effects are induced by these agents on combined treatment. These results support the view that combined therapies designed to target specific stages of biofilm maturation can be a most effective way to combat mycotic infections.

## MATERIALS AND METHODS

### Chemicals and Materials

Ketoconazole (KTC), amphotericin B (AMB), farnesol (FA) and tyrosol (TY) were from Sigma (MO, USA); they were dissolved in DMSO, 5 mg/ml, and stored at -20°C. Sabouraud dextrose agar and Nutrient agar were from LAB M (England). RPMI 1640 with 2% L-glutamine, Fetal Bovine Serum (FBS), Penicillin/Streptomicin solution and Trypsin EDTA 10X were from PAA (Austria). Collagen Type I from calf skin and collagenase were from MP Biomedical (OH, USA). Petri dishes, 6-well plates and 96-well plates were from Greiner Bio One (Austria).

### Microrganisms and Cells

*Candida albicans* strain 126, from the collection of the Institute, was selected among others because it displayed unique sensitivity to farnesol and tyrosol, in agreement to published reports [8,9]. The organism was maintained in Sabouraud dextrose agar plate. *Pseudomonas aeruginosa* ATCC 27853 was maintained in Nutrient agar plate, and it was used here solely for the production of conditioned media. In a preliminary co-culture study this microrganism was found to inhibit the growth of Candida, confirming a previous report describing a similar effect [10]. The human HSCO cells were isolated from a postirradiation oropharyngeal squamous carcinoma and retain the epithelial morphology after multiple passages in culture. The human HECV umbilical cord endothelial cells were purchased from ATCC (Rockville, MD, USA). Both cell lines were maintained in complete medium (RPMI 1640 medium enriched with 2% L-glutamine and 0.5% Penicillin/Streptomicin and supplemented with 10% FBS). They were incubated at 37°C in a humidified incubator (5% CO_2_, 95% air). Confluent cultures were harvested with trypsin-EDTA and passaged in monolayer.

### Preparation of Conditioned Media

Confluent HSCO and HECV cells were cultured in complete medium for 48 hours, after which time the media were harvested and filtered. *P. aeruginosa* was allowed to growth in RPMI 1640 with 2% L-glutamine and after 48 hours the bacterial suspension was centrifuged and filtered. All the conditioned media were stored at -20°C for future use.

### Collagen Gel Preparation

We followed the standard procedure described previously [11]. Briefly, the stock solution was prepared by dissolving 100 mg acid-soluble Type I collagen in 33 ml of sterile distilled water supplemented with 0.1% acetic acid. The final ready-to-use collagen solution was obtained from 1 volume of a 2:1 mixture of 10X RPMI 1640 and 0.34 M NaOH mixed with 4 volumes of the stock solution. This solution, the complete collagen mixture to serve as the 3-dimensional substrate for cell growth, does not gel if kept in ice.

### Growth in Collagen Gel

Candida cells were taken from agar, resuspended in the culture medium, counted and adjusted to the density of 1x10^3^ cells/ml. Following centrifugation, the pellets were resuspended in complete collagen solution, and a volume of 50 μl was dropped into each well of a 6-well plate. After harvest, HSCO and HECV cells were resuspended in complete medium, counted and adjusted to the density of 5x10^5^ cells/ml. Following centrifugation, the pellets were resuspended in the collagen solution, and a volume of 50 μl was dropped into each well of a 6-well plate. Control groups contained cell-free gels. The collagens were allowed to gelify in the incubator and after 40 min 4.0 ml of complete medium were added per well.

### Co-Culture Infections

After 6 days in collagen, HSCO- and HECV-containing gels were infected by mixing Candida cells in fresh complete medium at the density of 1x10^3^ cells/ml.

### Antifungal Susceptibility Testing

The Minimum Inhibitory Concentration (MIC) was determined by a standard CLSI broth microdilution method [12]. *C. albicans* strain 126 was shown to be resistant to KTC (MIC, 16 μg/ml) and susceptible to AMB (MIC, 0.25 μg/ml). We evaluated also the MIC value of farnesol and tyrosol and found it to be very high (256 and 1024 μg/ml, respectively).

### Treatments

Based on preliminary approaches and on the MIC values, the present experiments were conducted with the sub-inhibitory doses of 0.125, 8.0, 26.6 and 16.6 μg/ml, AMB, KTC, FA and TY, respectively. Treatments were generally conducted on 24-h-old cultures. However, in sessile Candida certain treatments were started at the stage of nascent colonies (5-hours-adhesion period). To probe the radiosensitivity of Candida, the cultures were exposed to γ-radiation, by delivering escalating single doses of 2, 8, 16 and 32 Gy by 1.2 MV (-photons (Theratron-780 Teletherapy unit -CTT) at 37° C. Based on these results, irradiation was set to deliver to cell plates a final total radiation dose of 8 Gy at the dose-rate of 0.90 Gy/min.

### Candida Survival

The survival of sessile and planktonic Candida, exspressed in Colony Forming Unit (CFU), was determined by the Viable cell count as reported by Miller [13]. In the sessile condition Candida cells were first released from the collagen gels by collagenase. Thereafter, samples of 100 μl/well of Candida suspension were conveniently diluted to obtain a density of 1x10^2^ to 1x10^3^ cells/ml to be plated on Sabouraud agar medium. After 24 hours the colonies were counted to calculate the number of candida, expressed as CFU/ml x10^6^.

### Statistical Analysis

The data were elaborated with the Graph Pad Prism statistical programme. The results are expressed as means ± SEM of triplicate incubations per group. At least three independent experiments were conducted to confirm results. Differences were considered significat when p<0.05, as assessed by the *t*-tests. Asterisks in the figures represent p values as follows: *, p<0.05; **, p<0.005; ***, p<0.0001.

## RESULTS

**Fig. (1):**
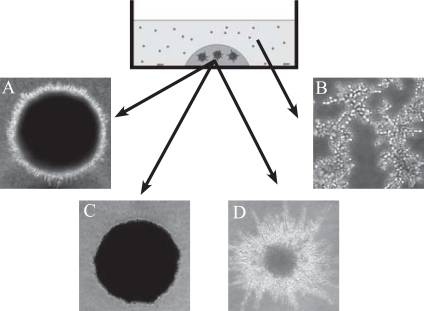
Schematic representation of the collagen gel model as used to growth Candida cells. Representative morphologies are: **A)** mature colony; **B)** planktonic yeast; **C)** yeast-colony and **D)** filamentous colony. Magnifications are: A, 40X and B, C and D, 20X.

The basic model serving the present experiments is outlined in Fig. (**1**), where examples of the main morphologies outlined below are also shown. When 1x10^3^ Candida cells/ml were embedded in collagen gel for a period of 24 hours, they typically originated 10 to 20 sessile multicellular colonies/gel, each consisting of a dense aggregate of yeast cells making up the core colony, bordered by multiple filaments composed of pseudohyphae and hyphae, radially distributed and covering an average area of 0.5 mm^2^ (Fig. **1A**). In many respects the morphology of these colonies resembled the biofilms described in published reports [14]. However, they were named “mature colonies”, because no effort was made to detect the possible presence of an extracellular matrix, a polymeric structure considered an hallmark of microbial biofilms [15]. In the co-culture experiments Candida cells were mixed in the culture medium, where they proliferated as free planktonic yeast organisms with occasional pseudohyphae (Fig. **1B**). As a general rule, Candida grew approximately 10 times more abundant during sessile as compared to planktonic condition. 

### AMB and KTC Exert Opposite Effects on Planktonic and Sessile *C. albicans*

The effects of 0.125 and 8.0 μg/ml AMB and KTC, respectively, on 24-hours-old cultures are shown in Fig. (**2A** and **B**).

**Fig. (2):**
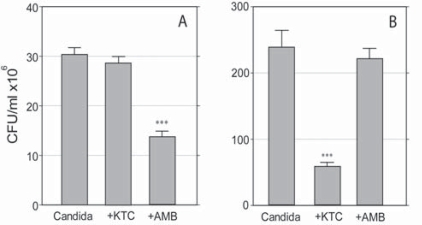
Effects of KTC and AMB on the survival of planktonic **(A)** and sessile **(B)** *C. albicans.* Differences in scale reflect the different growth rate displayed by the organism grown in free floating medium as compared to growth in collagen gel, respectively. (***,p<0.0001).

In the planktonic cultures CFU values were 30.6 ± 1.4 in the control group, and 13.9 ± 1.0 and 28.5 ± 1.2 in the AMB and KTC treatment groups, respectively. AMB, but not KTC, significantly inhibited the growth of planktonic Candida (p<0.0001) (Fig. **2A**). The opposite effect was observed with sessile Candida. Here, treatment with KTC significantly inhibited the growth of the organism, with a CFU value as low as 54.8 ± 5.8, down from the value of 225.0 ± 23.6 detected in the control group (p<0.0001). No significant damage was induced by AMB, with a CFU value of 220.0 ± 15.9 (Fig. **2B**). In addition, there was a distinct effect on colony morphology, in that KTC completely inhibited filamentation, leaving core colonies filled with yeast cells (yeast-colonies in Fig. **1C**); AMB induced a marginal shrinkage of the colonies and slightly inhibited filamentation.

### The Co-Culture with Human Cells Modulate the Response of Planktonic Candida to AMB and KTC

In preliminary experiments in which Candida cells were added to the culture medium of 6-day-old HSCO and HECV-containing collagen gels, it was consistently found that HSCO cells enhanced and HECV cells inhibited, the proliferation of planktonic Candida. The fungal morphology was also deeply affected, in that there were many filamentous phenotypes with HSCO cells but not with HECV cells, where filamentation was almost absent. We determined the effects of AMB and KTC on the survival of 24-hours-old planktonic Candida in these experimental conditions, as detected by the CFU procedure performed 24 hours after treatment. The results of this approach are shown in Fig. (**3**) as percent of the untreated control set at 100%.

**Fig. (3):**
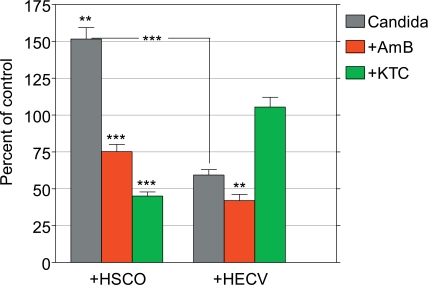
Percent effects of AMB and KTC on the survival of planktonic *C. albicans* in the presence of human HSCO and HECV cells.(**, p<0.005; ***, p<0.0001).

In the absence of the drugs the incidence of Candida co-cultured with HSCO cells increased to a value of 152% over the control group, while only 59% of them survived in the presence of HECV cells. The survival incidence in the groups with 0.125 μg/ml AMB was 75% and 42% Candida co-cultured with HSCO and HECV cells, respectively. Following treatment with 8.0 μg/ml KTC, the figures were 45% and 105%, respectively (Fig. **3**). When the capability of AMB and KTC to inhibit the survival of Candida was compared, it was found that in the several experimental conditions they diverged significantly (p<0.0001), and while the highest inhibitory effect of AMB was displayed on Candida co-cultured with HECV cells, the co-culture of Candida with HSCO cells was required to achieve maximal effect by KTC.

### Farnesol Modulates the Antimycotic Effects of KTC and AMB

These findings strongly suggested that filamentation was a morphological target of KTC, although not of AMB. To further explore this observation Candida cells were embedded in collagen gel at the density of 1x10^3^ cells/ml and 5 hours later they were exposed to 26.6 μg/ml FA, a quorum sensing factor known to inhibit the morphological transition of *C. albicans* and promote the growth of yeast forms [8]. After 24 hours from culture the gels were treated with 0.125 and 8.0 μg/ml AMB and KTC, respectively. The results are summarized in Fig. (**4**).

**Fig. (4):**
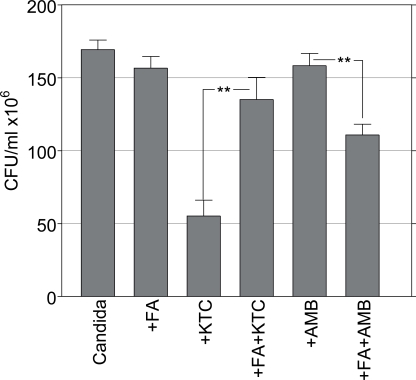
Effects of AMB and KTC on the survival of sessile *C. albicans* pretreated with FA. (**, p=0.002).

In control Candida there were morphologically mature colonies and a CFU value of 169.3 ± 6.5. After FA treatment mainly yeast-colonies were observed, although the survival was comparable to control, with a CFU value of 156.6 ± 8.0. The combined treatment of FA with KTC gave a CFU value of 135.1 ± 15.2, up from the CFU value of 55.2 ± 10.8 detected with KTC alone (p=0.002). By comparison, the combined treatment of FA with AMB resulted in a CFU value of 110.8 ± 7.4, significantly lower than the CFU value of 158.3 ± 8.4 in the group with AMB alone (p=0.002) (Fig. **4**). This finding, that the absence of filamentous phenotypes was associated with a reduced effect of KTC and an enhanced effect of AMB, underline the importance of morphological transitions as targets of the pharmacodynamic activity of antimycotic drugs during Candida infection.

### *C. albicans* can be Modulated to Respond to γ-Radiations

To probe the radiosensitivity of *C. albicans*, Candida cells were grown in planktonic and sessile conditions and after 24 hours they were exposed to γ-radiations, by delivering escalating single doses of 4, 8, 16 and 32 Gy. After 24 hours the survival of Candida was determined by the CFU procedure. Fig. (**5**) shows the results of this approach as percent of control set at 100%.

The survival incidence of both sessile and planktonic Candida was comparable to control in the several treatment groups, except the group of sessile Candida treated with 32 Gy, with a survival incidence of 74%, significantly lower compared to the control (p<0.01). This experiment demonstrated that *C. albicans* strain 126 is fairly radioresistant to γ-radiation, considering that the dose of 32 Gy is comparatively far above the doses used in radiotherapy. Based on these results, all the remaining experiments were conducted by using the radiation dose of 8 Gy, regarded in the range of clinical exposure. Next, HSCO and HECV cells were embedded in collagen gel and 6 days later they were infected with Candida, 1x10^3^ cells/ml, mixed in the culture medium. After 24 hours the cultures were irradiated with 8 Gy and after another 24 hours Candida survival was detected by the CFU method. The results are presented in Fig. (**6**).

The CFU values of Candida cultured alone were 24.4 ± 1.5 and 21.8 ± 0.5, untreated and radiation-treated groups, respectively. The matching figures in the groups co-cultured with HSCO cells were 35.4 ± 1.9 and 22.2 ± 1.6 and in the groups co-cultured with HECV cells were 12.2 ± 0.9 and 17.6 ± 1.5, respectively. There was a highly significant difference among the unirradiated groups compared to control Candida alone, with HSCO cells enhancing and HECV cells inhibiting the proliferation of Candida (p<0.0001 in both cases). Under the effects of irradiation these values changed dramatically, and while Candida proliferation was inhibited in the co-culture with HSCO cells (p<0.0001) it was increased in the co-culture with HECV cells (p=0.004) compared to the matched unirradiated groups.

To further confirm these results, Candida cells were embedded in collagen gel and after 5 hours (stage of nascent colonies) they were divided in 4 groups and treated as follows: group I) control; Group II) Conditioned medium from HSCO (CMHSCO) cells; group III) Conditioned medium from HECV (CMHECV) cells and group IV) Conditioned medium from P. aeruginosa (CMPa). All the conditioned media were diluted 1:2 in the culture medium. For comparative purpose, two additional groups were treated with FA (group V) and TY (group VI), mixed in the culture medium at the equimolar dose of 0.12 mM (26.6 and 16.6 μg/ml, respectively). The results are shown in Table 1. The culture conditions affected the morphology of Candida in that after 24 hours there were mature-colonies in group I, filamentous colonies (see Fig. **1D**) in groups II, IV and VI and yeast-colonies in groups III and V. The morphologies observed in groups V and VI were in agreement with reported criteria [16]. Upon confirmation of the morphologies, a set of these groups was left untouched and a set was irradiated once with 8.0 Gy. After 24 hours from irradiation Candida survival was detected by the CFU method (Table 1). The survival of Candida in the unirradiated groups was 186.7 ± 11.4, 163.3 ± 20.6, 96.7 ± 5.6, 11.7 ± 1.3, 184.3 ± 2.1 and 143.4 ± 5.5 in groups I, II, III, IV, V and VI, respectively. The matched values of CFU in Radiation-treated groups were 178.3 ± 14.7, 90.0 ± 11.5, 90.0 ± 16.5, 2.1 ± 0.6, 177.2 ± 6.0 and 62.2 ± 8.1, respectively. The induction of filamentation caused by CMHSCO, CMPa and TY was associated with an increased radiosensitivity, as shown by the survival inhibition of Candida in the groups II, IV and VI compared to the unirradiated counterparts (p=0.01, p<0.0001 and p<0.0001, respectively).

### Synergistic Effects of KTC and γ-Radiations

The above results indicated that during filamentation C. albicans becomes sensitive to damage by γ-radiations in a way similar to KTC. Given this premise, we speculated that under specific experimental conditions γ-radiations might act synergistically with KTC in inhibiting Candida survival. To examine this possibility, HSCO cells were embedded in collagen gel for 6 days, after which time they were infected with Candida in the culture medium. The presence of HSCO cells caused planktonic Candida to turn into filamentous forms. There were 4 experimental groups as follows: 1) Control; 2) Treatment with KTC, 8.0 μg/ml, after 24 hours from infection; 3) Irradiation with 8.0 Gy after 48 hours from infection and 4) Sequential treatment with KTC and γ-radiations. For comparative purpose, 4 groups with planktonic Candida cultured alone were treated in the same way. The survival of Candida was determined by the CFU procedure after 24 hours from the last treatment, and the results are shown in Fig. (**7**). 

Candida cultured alone had CFU values of 28.8 ± 2.1, 34.8 ± 3.3, 28.4 ± 1.0 and 26.1 ± 0.7 in untreated and KTC-, radiation- and combined KTC plus radiation-treated groups, respectively. This compares to CFU values of 50.3 ± 3.7, 35.3 ± 3.9, 27.8 ± 1.3 and 15.0 ± 0.8, respectively, in the matched groups of Candida co-cultured with HSCO cells. The proliferation of Candida was greatly enhanced by the co-culture with HSCO cells, compared to Candida alone (p<0.0001), as was filamentation. In Candida cultured alone, the several treatments yielded CFU values that were comparable to the untreated control. Following the co-culture with HSCO cells, the sensitivity of Candida to treatments was increased and its survival generally impaired. Specifically, compared to the matched control, KTC alone significantly inhibited the survival of Candida (p = 0.01), as did radiations alone (p<0.0001). The sequential treatment with KTC and radiations further inhibited the survival of Candida, and this effect was synergistic when compared to the groups with KTC or radiations alone (p<0.0001) (Fig. **7**).

## CONCLUSIONS

In patients affected by candidiasis Candida cells display two main phenotypes, depending on whether they adhere to mucosal surfaces or migrate with the bloodstream. These phenotypes are similar to the sessile and planktonic phenotypes observed during growth in collagen gel and in the culture medium as described above, respectively. Specifically, the growth in collagen gel resulted in highly organized biofilm-like colonies, showing a variety of cellular transitions from yeast forms to hyphae, of the kind described in infected medical devices [17]. In comparison, planktonic Candida were much less heterogeneous, being comprised almost entirely of yeast forms. in the 3-dimensional context of the collagen gel Candida grew faster, roughly halving the generation time, as compared to Candida grown in floating culture media, vigorously achieving colony maturation within 24 hours of culture. This accelerated proliferation was associated with typical morphological transitions displayed by Candida in collagen gel, a behaviour likely to mimic the response of Candida to host tissues during infective invasion.

The pharmacodynamic properties of AMB and KTC have been extensively investigated using mainly free-floating (planktonic) Candida [4]. However, their effects on the sessile conditions that are encountered in many biofilm-associated infections may be not adequately addressed by these approaches, especially since Candida biofilms display a cellular heterogeneity reminiscent of multicellular organisms and are more resistant to treatments compared to free-floating cells [18]. In the present model system divergent effects were induced by AMB and KTC, in terms that while AMB was maximally effective in inhibiting planktonic Candida, KTC actively inhibited the survival of sessile Candida. Since filamentation is an integral component of *C. albicans* maturation and a driving force behind colony formation in collagen gel, a probable clue to these results is the observation that KTC, but not AMB, suppressed specifically the filamentous phase of sessile Candida. On the contrary, yeast forms were the target of AMB, thus making sense of the present results that AMB was highly effective on planktonic growth, but displayed poor antimycotic activity on collagen gel colonies. Our results seem to be at odd with previous reports on the effects of AMB and KTC on planktonic and sessile forms of *C. albicans* [19]. Essentially, these studies suggested that AMB is more likely to act by inhibiting the morphogenetic transformation of *C. albicans,* while KTC is more likely to block growth of budding yeasts. However, their model differed fondamentally from the present one, in that AMB and KTC were not assayed in mature colonies, where morphology-dependent toxicity can be evaluated against the architectural and phenotypic properties of Candida [20]. Consistent with this view are a number of reports, including the observation that yeast and hyphal forms have different levels of DNA methylation [21], and the more recent identification of an increased content in β-1,3 glucan in the cell wall of sessile compared to planktonic Candida [22]. These morphogenetic transformations are by no means marginal to Candida-host interactions, since recent studies have indicated that macrophages recognize yeast but nor filamentous forms in a process mediated by the protein dectin-1, and that yeast but not hyphae induces apoptosis of MCF-7 mammary carcinoma cells [23,24].

The mechanisms by which HSCO cells enhanced both the proliferation and filamentation of *C. albicans *and, on the contrary, HECV cells inhibited these phenotypes, leaving only sparse yeast aggregates, are not known. The morphology induced by the co-culture with HSCO cells would be consistent with the enhanced secretion of tyrosol by Candida, a filament-inducing quorum sensing molecule known to support unidirectional yeast to hyphal transitions in Candida biofilms [9]. On the other hand, the yeast forms induced by the co-culture with HECV cells would fit with the enhanced secretion of farnesol by the fungus. The effects of this quorum sensing molecule are indeed associated with the inhibition of filamentation and the promotion of planktonic yeasts [25]. While Candida-enhanced secretion of farnesol and tyrosol may have partially contributed to the observed effects, another input was probably provided directly by HSCO and HECV cells, a possibility that would fit a number of reports dealing with the interaction of *C. albicans* with epithelial and endothelial cells [26,27]. Although many uncertainties remain on the cellular components and pathways involved in this interaction, in fact, there is a general consensus that the encounter with Candida species stimulates epithelial and endothelial cells to internalize the organism by endocytosis, a process widely regarded as an important step in the defense system mounted by the host [28]. We did not investigate this topic, although our model would imply that soluble, rather than membrane-bound factors, were mainly responsible for the present results, as confirmed by the effects of HSCO- and HECV-conditioned media on the survival of sessile Candida (Table **1**).

**Fig. (6):**
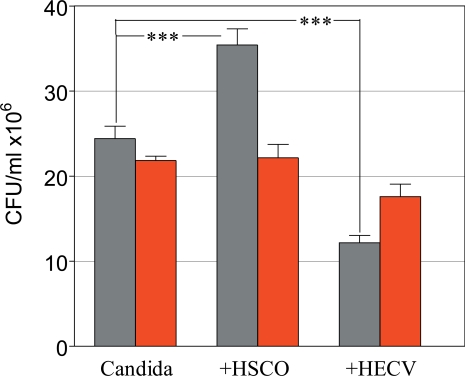
Role of HSCO and HECV cells in modulating the radiosensitivity of planktonic Candida. Survival of Control (gray) and Irradiated (red) *C. albicans.* (***, p<0.0001).

**Fig. (7):**
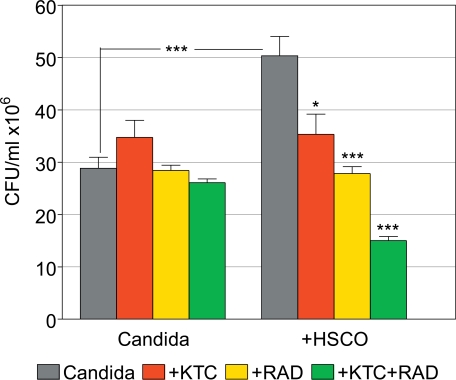
Synergistic effects of gamma-radiations and ketoconazole on the survival of planktonic *C. albicans* in the presence or absence of HSCO cells. (*, p=0.01; ***, p<0.0001).

*C. albicans*, like many fungal pathogens, is generally resistant to ionizing radiations, probably resulting from a radioprotective effect of the biofilm matrix or by other protective means [29]. Therefore, it was of medical interest to know how radiations affected Candida in our model system. Specifically, we investigated how γ-radiations, at doses of up to 32 Gy, influenced the growth of *C. albicans* in both sessile and planktonic conditions, as assessed by growth in collagen gel and in free floating culture medium, respectively. It was found that neither condition was affected by this exposure in terms of cell proliferation or dimorphism, with the exception of sessile Candida that were significantly inhibited by treatment with 32 Gy. Considering that this high dose is far behind clinically relevant doses, substantially our approach confirmed previous reports on the resistance of *C. albicans* to ionizing radiation [5]. Knowing that Candida cells are frequently in contact with mucosal and vascular tissues, we determined the possible modulatory effects of epithelial and endothelial human cells on the radiosensitivity of *C. albicans *exposed to 8 Gy, a dose selected because its clinical relvance. The results of this approach were striking in that radiation-induced inhibition of Candida was found to depend on filamentous rather than yeast forms, as observed in the co-culture with HSCO and HECV cells, respectively (Fig. **6**). Such an effect was confirmed by experiments in which conditioned media from HSCO and HECV cells and from *P. aeruginosa*, another filament inducing agent, were used in place of the cells (Table **1**). This preference of ionizing radiations for Candida transitional phenotypes was not reported before, and may have an epigenetic basis, of the kind as implicated by the radiation-induced bystander effect [30].

The main conclusions concerning radiation effects are summarized in Table **2**. In general, the agents that induced filamentation (CMHSCO, CMPa and TY) were associated with an increased radiosensitivity of Candida, while no radiosensitivity was observed with the filament-inhibiting agents (CMHECV and FA). The only exception was represented by KTC. The inhibition of filamentation by this drug, in fact, was associated with an increase in radiosensitivity, as shown by the synergistic decrease in the CFU value, as compared to the CFU values in the groups treated with the single agents (Table **2**). While we have no explanation for this outcome, experiments in which the combined treatments were reversed (i.e., radiations followed by KTC) gave similar results (data not shown). Since filamentation in C. albicans is a multistep process, our finding would imply that both radiations and KTC targeted the same morphological stage.

Our model, whereby sessile Candida can reorganize into multicellular colonies complete with yeast and filamentous morphologies, bears some similarity with the formation of biofilms *in vivo* by way of a common scaffold, represented by collagen fibers. Collagens are major components of the body, and whenever medical devices are implanted in organs and tissues, collagens are likely to provide the most proximate host support on which Candida aggregates into mature biofilms. In Candida the dimorphic transition from yeast to hyphal phase is a crucial step in the formation of a biofilm, and mutant strains defective in the ability to germinate are unable to form mature biofilms and fails to deliver virulent factors in mice [31]. Therefore, the inhibition of filamentation seems to be a logical primary target for the antimycotic treatment of Candida. This sounds reasonable since under pathological conditions *C. albicans* is known to evolve into heterogeneous biofilms, consisting of microenvironments conducive to formation of resistant subpopulations. We found that a group of such cellular subpopulations, that comprising the filamentous phase of colony maturation, is particularly sensitive to the synergistic effects of ketoconazole and γ-radiation. While it is still debatable whether the responsibility for Candida virulence rests on yeast or filamentous forms [32] our data suggest that radiation treatment, in combination with filament-targeting drugs, may help further the clinical control of candidoses.

## Figures and Tables

**Fig. (5):**
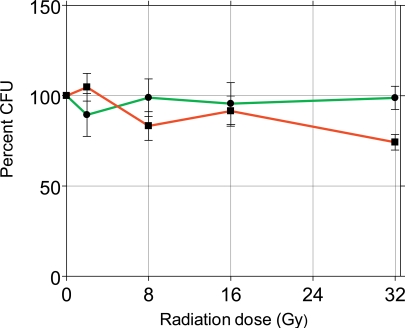
Percent effects of γ-radiations on the survival of sessile (red) and planktonic (green) *C. albicans.*

**Table 1.** Effects of Induced Filamentation on the Radiosensitivity of Sessile *C. albicans.* Treatments Included Farnesol and Tyrosol and the Conditioned Medium from HSCO (CMHSCO) and HECV (CMHECV) Cells and from *P. aeruginosa* (CMPa)

Group	Treatment	Morphology	CFU/ml x 10^6^±SEM		p value
			Control	+ RAD	
I	Untreated	Mature colonies	186.7 ± 11.4	178.3 ± 14.7	not significant
II	CMHSCO	Filamentous colonies	163.3 ± 20.6	90.0 ± 11.6	p=0.01
III	CMHECV	Yeast-colonies	96.7 ± 5.6	90.0 ± 16.5	not significant
IV	CMPa	Filamentous colonies	11.7 ± 1.3	2.1 ± 0.6	p<0.0001
V	Farnesol	Yeast-colonies	184.3 ± 2.1	177.2 ± 6.0	not significant
VI	Tyrosol	Filamentous colonies	143.4 ± 5.5	62.2 ± 8.1	p<0.0001

**Table 2.** Summary of the Effects of Ketoconazole, Farnesol, Tyrosol and the Conditioned Medium from HSCO(CMHSCO) and HECV (CMHECV) Cells and from *P. aeruginosa* (CMPa) on the Modulation of Radiosensitivity of Sessile *C. albicans*

Treatment	Filamentation	Radiosensitivity
Ketoconazole	Inhibited	Yes
Farnesol	Inhibited	No
Tyrosol	Enhanced	Yes
CMHSCO	Enhanced	Yes
CMHECV	Inhibited	No
CMPa	Enhanced	Yes
